# Using Experience Sampling Methodology Data to Characterize the Substance Use of Youth With or At-Risk of Psychosis

**DOI:** 10.3389/fpsyt.2022.874246

**Published:** 2022-05-03

**Authors:** David M. Weiss, Elizabeth Bernier, Douglas R. Robbins, Katherine M. Elacqua, Kelsey A. Johnson, Kate Powers, Raquelle I. Mesholam-Gately, Kristen A. Woodberry

**Affiliations:** ^1^Maine Medical Center Research Institute, MaineHealth, Portland, ME, United States; ^2^Department of Psychiatry, Tufts University School of Medicine, Boston, MA, United States; ^3^Massachusetts Mental Health Center Public Psychiatry Division of the Beth Israel Deaconess Medical Center, Boston, MA, United States; ^4^Department of Psychiatry, Harvard Medical School, Boston, MA, United States

**Keywords:** psychotic-spectrum disorders, ecological momentary assessment, early psychosis, psychotic symptom, negative affect, dynamic association, momentary data

## Abstract

**Objectives:**

Psychotic-spectrum disorders emerge during adolescence and early adulthood, which corresponds with the peak period for substance use initiation. Clinical and epidemiological data provide support that substance use is associated with psychotic symptom onset and severity. Experience-sampling methodology (ESM) data may provide additional insight into dynamic associations between substance use and psychotic symptoms. This is one of the first efforts to characterize substance use frequency and dynamic associations with psychotic symptoms and negative affect from ESM data in both clinical high risk (CHR) and early psychosis (EP) individuals.

**Methods:**

Using ESM, 33 individuals, including 17 with CHR and 16 EP (age range: 15–24), provided information on substance use, negative affect, and psychotic symptoms 6 times a day across a 21-day data collection window. Psychotic symptoms and negative affect included multi-item variables rated on a seven-point Likert Scale. Participants reported recent substance use for 4 drug classes (nicotine, cannabis, depressants, stimulants) via a yes/no item. Descriptive information included data on substance use frequency, and momentary negative affect and psychotic symptoms. Exploratory analyses included multi-level and person-level dynamic structural equation models, which assessed contemporaneous and lagged associations between substance use and symptoms.

**Results:**

Twenty-seven individuals (82%) reported recurrent substance use including stimulants (*n* = 12, 46%), nicotine (*n* = 9, 27%), cannabis (*n* = 6, 18%), and depressants (*n* = 4, 12%). Individuals with any recurrent substance use indicated usage at 47.7% of answered prompts; stimulants at 23.6%; nicotine at 74.2%; cannabis at 39.1%; and depressants at 20.1%. A multi-level dynamic structural equation model reflected that substance use (any class) was associated with lagged negative affect (β = −0.02, CI: −0.06, < -0.00) but no significant contemporaneous or lagged associations between substance use and psychotic symptoms. Person-level models suggest potentially meaningful inter-individual variability.

**Conclusions:**

CHR and EP individuals use a range of substances that may both reflect and influence other experiences in daily life experiences. Data reflected moderate to high rates of recurrent substance use with more consistent use within nicotine and cannabis classes. ESM data have the potential to increase our understanding of the dynamic relationships between substance use and symptoms and to inform treatment for individuals in early course psychosis.

## Introduction

Individuals with psychotic spectrum disorders are more likely to have substance use disorders compared to the general population (1, 2). Studies report elevated rates of substance use in individuals at-risk of psychosis or with first-episode psychosis across substance categories, including cannabis (42–54%), nicotine (16–75%), alcohol (17–44%), and stimulants (7–45%) (1, 3–6). Developmentally, psychotic-spectrum disorders emerge during adolescence and early adulthood, a period of time that overlaps with the peak ages for substance use initiation (7). Given this timing and the considerable comorbidity of substance use and psychotic disorders, greater understanding of the associations between substance use and psychotic symptoms is needed to help guide treatment, especially during the early stages of psychosis.

Prior studies suggest that (1) substance use contributes to earlier onset of psychosis and worsening of psychotic symptoms (8, 9), (2) substance use emerges subsequent to psychosis as a consequence of neurobiological changes or as a coping strategy to alleviate symptoms (10, 11), (3) adverse childhood experiences are related to the later onset of both substance use and psychotic disorders (12), and (4) shared genetic liabilities underlie both psychotic spectrum disorders and substance use (13–15). Understanding potential associations between substance use and psychosis is complicated by the fact that associations may differ by substance use class or by poly-substance use.

Conceptually, psychotic disorders develop in stages, including a premorbid stage with subtle challenges in cognition and functioning (16), the clinical high risk (CHR) stage with subthreshold psychotic symptoms (17), the first episode of psychosis with onset of acute psychotic symptoms, and the residual phase that may include decreased symptom severity and frequency of acute psychotic episodes (18). While some research suggests that substance use contributes to the onset of psychosis (early psychosis, EP), a systematic review has documented more studies with null findings than studies with non-null results (4). Though researchers often examine the relationship between psychosis and substance use onset across all substance classes, a substantial proportion of studies focus specifically on cannabis use and psychotic disorder onset. Multiple studies indicate a correlative relationship between cannabis use and age of onset (8, 19–21), including evidence to suggest a dose-response relationship between levels of cannabis use and psychosis risk (22), though others report null findings (1).

While the evidence supporting the link between substance use and psychotic disorder onset has been mixed, the evidence supporting the link between substance use and greater psychotic symptom severity is more consistent. Baseline data of the Recovery After an Initial Schizophrenia Episode Early Treatment Program (RAISE-ETP), the NIH multisite trial that helped establish coordinated specialty care as the predominant model of care for early psychosis in the U.S. (23), support the association of substance use to more severe symptoms within individuals with early psychosis (24). Similar findings indicate cannabis use is associated with more severe symptoms (25, 26).

One major limitation of the existing literature on substance use and psychotic disorder associations is that it is based largely on epidemiological and clinical data from cross-sectional studies or longitudinal studies with a small number of measurements over months or years. Associations are either contemporaneous or between retrospectively classified substance use and symptom patterns. There are very limited intensive longitudinal data that examine patterns of substance use patterns and psychotic symptoms on a day-to-day or within-day basis, the time frame of expected bidirectional influence and strongest association. Thus, clinicians and researchers alike have limited understanding of the degree to which substance use may influence the day-to-day experiences of psychotic symptoms or vice versa.

Experience-sampling methodology (ESM) data may provide important insights into dynamic associations between substance use and psychotic symptoms. Methodologically, ESM has individuals track moment-to-moment symptoms (27, 28), an important sampling strategy considering the variable and episodic nature of psychotic disorders and substance use. Use of ESM designs may be particularly useful to evaluate the “self-medication hypothesis (29)” and the “reward deficiency syndrome (30)”, theories that posit that youth in the high-risk or early stages of psychotic disorders use substances to attenuate emerging, and often brief or momentary, symptoms (10). However, previous ESM research is limited to two studies that examined cannabis associations in adult samples with psychotic disorders (31, 32). No ESM studies have assessed broader categories of substance use and symptom associations, particularly in an adolescent/young adult sample with CHR or EP.

To address this gap, we performed secondary analyses on ESM data from a study that examined the degree and temporal variability of affect, psychotic spectrum symptoms and thoughts of self-harm over the course of 21 days among youth at CHR for psychosis or with EP. The goals of these secondary analyses were to characterize substance use at a day-to-day and within-day level and explore the temporal within-person relationships between substance use, negative affect (NA), and psychosis.

## Methods

### Participants

**Figure 1:**
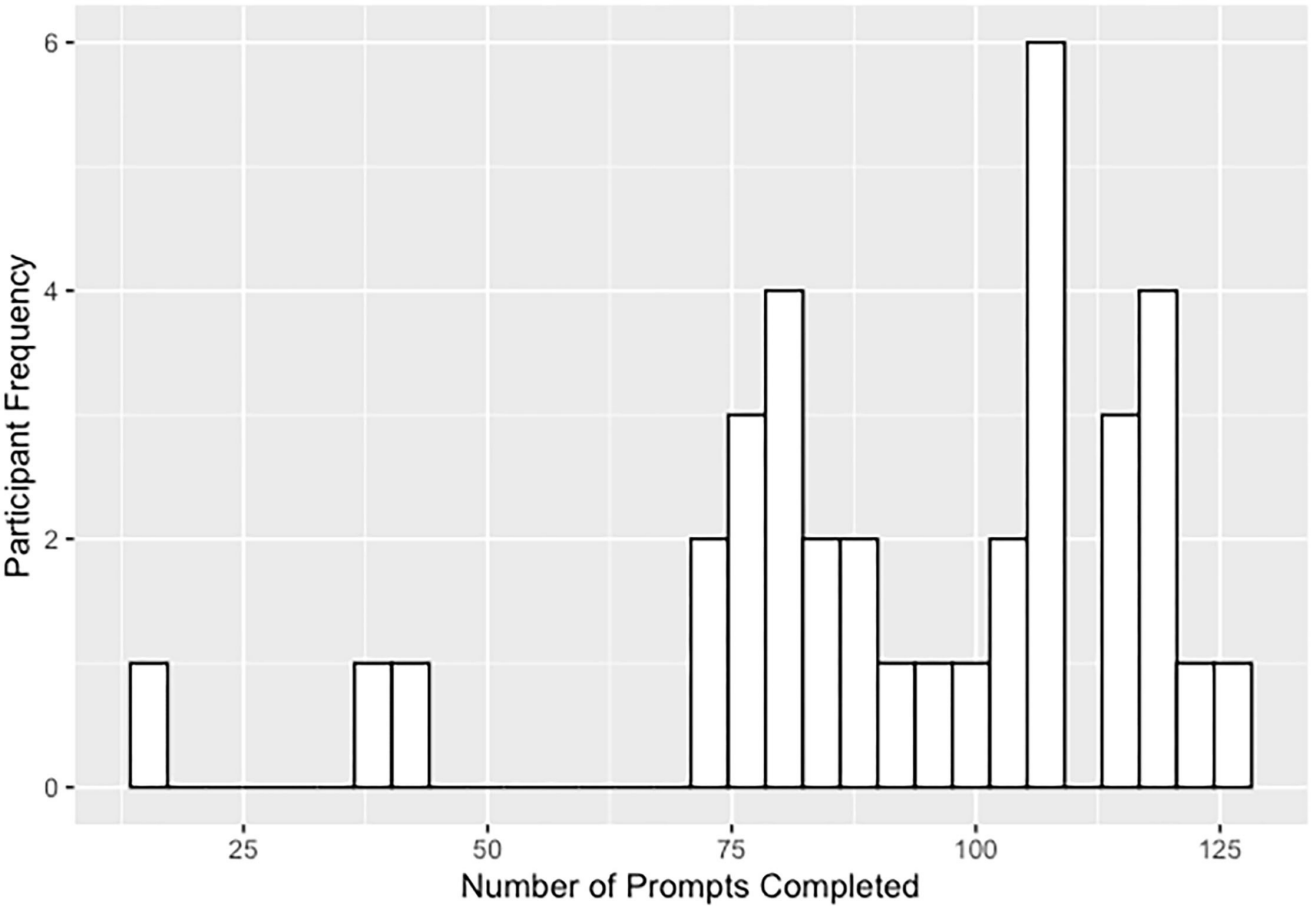
Distribution of answered prompts for the 3-week ESM study including the 3 participants excluded due to low number of answered prompts.

Experience-sampling data originated from a dataset of 69 participants including 36 individuals on the psychotic spectrum and 33 healthy controls. Data from 33 psychotic spectrum participants (51.5% CHR, 48.5% EP, including both affective and non-affective psychotic disorders) were included in the present study; three participants were excluded due to the low number of answered prompts (e.g., more than two standard deviations below the mean number of answered prompts; see Figure 1). Two participating sites at Maine Medical Center (MMC) and Beth Israel Deaconess Medical Center (BIDMC) recruited participants over a year and half. These sites have research and clinical programming and established referral networks for those with psychotic spectrum disorders. Eligible participants (1) were between the ages of 15 and 25, (2) spoke fluent English, (3) had an estimated IQ above 70, and (4) were willing and able to complete ESM procedures. Participants were ineligible if they had a current comorbid medical, neurological, or moderate to severe substance use disorder that would likely have a confounding impact on affect or psychotic symptoms. Baseline diagnostic assessments, conducted by trained clinician interviewers, determined if the participants met one of the following: (1) Criteria of Psychosis-Risk Syndromes (COPS) (within the 6 months prior to their participation) or currently meeting criteria for Attenuated Positive Symptom or Brief Intermittent Psychotic Syndromes, Persistent, based on the Structured Interview of Psychosis-Risk Syndromes [SIPS; Miller et al. (33)] or (2) criteria for a DSM-5 (34) psychotic-spectrum disorder, including schizophrenia-spectrum and mood disorders with psychotic symptoms but excluding substance-induced psychotic disorders.

### Procedures

This multisite ESM study examined behavioral self-report data collected using the smartphone application MetricWire (www.metricwire.com), a HIPAA-compliant commercial service that sends surveys to participants' phones throughout the course of their daily lives. Institutional review boards at BIDMC and MMCRI approved study procedures. Research staff recruited participants using various forms of digital announcements and flyers and by referrals from community partners. Written informed consent was obtained from all adult participants. Written consent of a legal guardian and written assent were obtained for participants under the age of 18. Study staff provided participants with an orientation to the MetricWire app and survey, with instructions on how to respond to surveys at 6-semi random prompts per day for 21 days. Staff instructed participants to respond to each survey prompt “in the moment” and to respond as soon as possible after receiving the prompt. Participants received six daily prompts, one per each 2-hour time block within a 12-hour window Pre-selected by the individual to be during typical waking hours (*e.g*., 9am−9pm). Prompts were considered semi-random as participants received prompts at random times during each 2-hour time block in an effort to limit anticipatory responses. They were provided with a 15-minute window to respond to prompts. Participant remuneration was provided weekly for a minimum response rate of 50% and included weekly bonuses for high total responses, including extra bonuses for sustained compliance across all 3 weeks. Participants could earn up to $180 for the ESM component of the study.

### Measures

Demographic information questionnaire. Collected information on the participant's age, sex at birth, gender, race, ethnicity, occupation, living arrangement, and the education and income of the participant and their legal guardian.

#### Diagnostic Assessments

Structured clinical interview of DSM-5 select Axis I & Axis II Disorders (*SCID-5RV, Research version)*; (34), is the leading interview for assessing disorders from the Diagnostic and Statistical Manual of Mental Disorders (35). The following modules were administered: Schizophrenia Spectrum, Bipolar, Substance-Related and Addictive Disorders, and Depressive Disorders. Anxiety and Trauma- and Stressor-Related sections were administered only upon relevant positive SCID-5RV screens. When applicable, staff secured written permission to communicate with family members to elicit additional information (observations and treatment, developmental, and family histories).

Structured Interview of Psychosis-Risk Syndromes [*SIPS, version 5.6;* Miller et al. (33, 36)], is one of two internationally accepted and validated interviews for assessing putatively prodromal symptoms and syndromes. Interviewers administered positive symptom queries to all participants who did not meet the criteria for a psychotic disorder.

#### Experience Sampling Methodology Variables

Substance use, NA, and psychotic symptom queries were embedded in a longer set of items asking participants about their momentary positive mood and social context. Instructions guided participants to answer each item relevant to the specific moment in which the phone prompt occurred to capture momentary rather than retrospective data. Only a small number of select items, including those about substance use, inquired about events since the last prompt, and these items were asked after momentary items to minimize the influence of retrospective thinking on momentary ratings.

##### Substance Use

Substance use was assessed at each prompt with one question: “Since the last beep [prompt], I have used/taken…” The participants were then given eight options, with examples, to select including: Depressants (ex. alcohol, xanax, klonopin, ativan), Stimulant or Caffeine, Sedatives (allergy or sleep medicine, oxycontin), Psychedelics (ex. LSD, ecstasy), Nicotine (ex. cigarettes, tobacco, or vaporizers), Cannabis, Other, and Nothing. Staff staff instructed participants to include prescription and over-the-counter medications or substances as well as illicit substances for the given classes. One item, “In the past 24 hours, did you take your prescribed medications” was included during the first answered prompt of the day with the following responses: “All, as prescribed”, “Yes, but not all as prescribed”, “No”, and “I have no prescription medications”.

##### Negative Affect

Negative affect (NA) was measured by responses to 6 items, each beginning with the stem “I feel” and followed by descriptors (“irritable”, “lonely”, “down”, “insecure”, “guilty”, “stressed”) (37). These were interspersed with 3 items assessing positive affect (“happy”, “relaxed”, “content”). Participants reported how strongly they felt each emotion at that moment based on a 7-point Likert scale from 1 (“Not at all”) to 7 (“Very much”). Momentary NA was calculated as the mean response for all 6 items answered at each prompt. The 6-item NA scale showed satisfactory within-person and between person reliability (ω = 0.73, ω = 0.93, respectively). To account for between-person variability on mean levels of NA, deviations from the individual's person-centered mean over the course of the study were used in exploratory modeling (38).

##### Psychotic Symptoms

Psychotic symptoms were evaluated by responses to 9 items: “I feel suspicious”, “I can't let go of my thoughts”, “My thoughts are influenced by other people”, “I feel unreal”, “I see things that other people can't see”, “I hear things that other people can't hear”, “I feel like I am losing control (of my thoughts)”, “It's hard to express my thoughts in words”, and “My thoughts are so loud it's as if I can hear them” (37). These responses reported the degree to which the participants experienced each item at the moment of the prompt. Responses were recorded on 7-point Likert scale from 1 (“Not at all”) to 7 (“Very much”). Momentary psychotic symptoms was calculated as the mean response for all 9 items answered at each prompt. The 9-item scale showed satisfactory within-person and between-person reliability (ω = 0.78, ω = 0.96, respectively). To account for between-person variability on mean levels of psychotic symptoms, deviations from the individual's person-centered mean over the course of the study were used in exploratory modeling (38).

### Statistical Analysis

We categorized a participant as having recurrent substance use (RSU) if they indicated substance use during at least 5% or more answered prompts. In determining recurrent substance use, means and standard deviations for each substance class were examined for participants who indicated use for a specific substance class during 2 or more prompts. After examining the lowest values within one standard deviation of the mean for each substance use class, the 5% of prompts cutoff was selected as the standard to separate infrequent or occasional use from RSU across all substance use classes. Descriptive data and summary statistics (39) were used to characterize the rate of RSU in each substance class, the percent of prompts reflecting use, and the frequency of use across multiple classes. Diagnostic group differences (*i.e*., CHR and EP) were evaluated via chi-square tests for categorical variables and *t*-tests for continuous variables and *post hoc* power analyses were conducted with G^*^Power Version 3.1 (40). Visualizations of prompts and substance use frequencies were created using the package “ggplot2” (41) and “plotly”(42) in R version 3.6. Within-person and between-person reliability omega coefficients were calculated using the R package “multilevelTools” (43).

**Figure 2:**
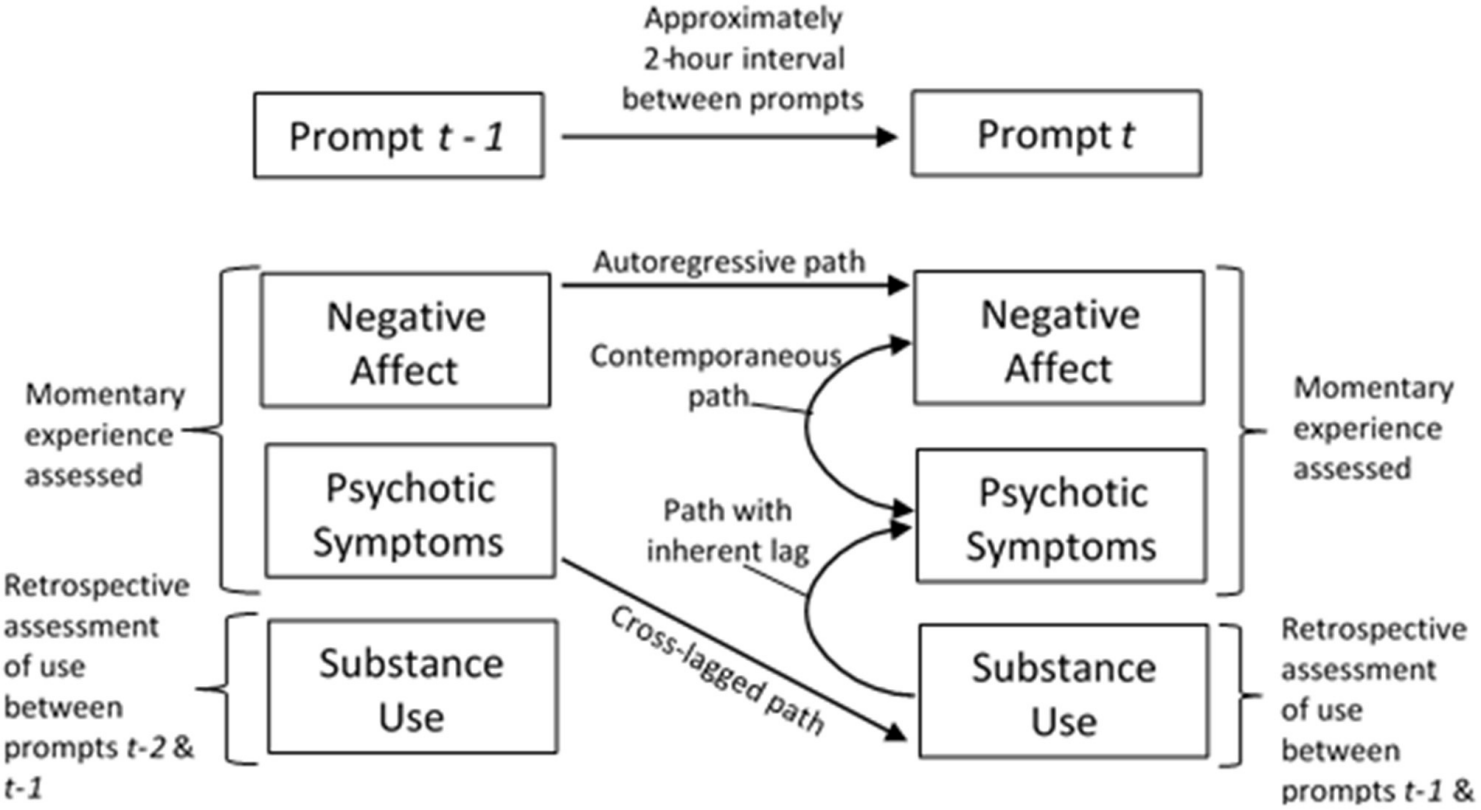
Experience sampling methodology with multilevel vector autoregressive (MVAR) estimation of autoregressive, cross-lagged, and contemporaneous paths.

Exploratory multilevel vector autoregressive (MVAR) models estimated within the dynamic structural equation modeling framework in Mplus 8.3 (44) used person-centered means of psychotic symptoms and NA in addition to a binary substance use indicator variable (any class of substance). MVAR were used in the analyses of ESM data as these models can accommodate the two-level structure of ESM data, the use of multiple outcomes, and estimation of contemporaneous (*i.e*., 0-lag) *and* lagged relationships, which measure the extent that a symptom at timepoint (*i.e*., prompt) *t*-1 predicts itself at timepoint *t* (38). For the current study, we were particularly interested in whether substance use influenced deviations from participants' person-centered means of psychotic symptoms and/or NA. Cross-lagged and contemporaneous regression paths provide an indication of whether substance use exacerbates or reduces one's experience of psychotic symptoms and negative affect at the previous prompt or at the current prompt, respectively (Figure 2). Given the retrospective manner in which the substance use question was asked (e.g., “Since the last prompt…”), there is a lag embedded in what would otherwise be considered a contemporaneous path between substance use and psychotic symptoms or NA. Parameter estimates were considered to be significant if the 95% credible interval did not include 0. The MVAR model allowed within-person residuals to vary across individuals; the log of residual variances was estimated to ensure that all residual variances are positive.

The MVAR model was limited to analysis of substance use (any class) rather than specific substance use classes due to sample size limitations for multi-level modeling. In recognizing that substance use associations to psychotic symptoms and NA may vary the individual or substance use class, we estimated individual autoregressive (1) lag models for each participant who indicated substance use. These were conducted to assess whether between substance use associations to psychotic symptoms and negative affect varied from the within-person associations estimated in the MVAR model. For both sets of models, time was transformed into discrete 2-hour intervals to accommodate existing ESM procedures (semi-random prompts delivered during 6 2-hour blocks) using the “TINTERVAL” function in Mplus. This was performed to allow for the unequal spacing between measurements that naturally occur between days (e.g., accounting for non-measurement intervals that occur during sleep) and for days in which fewer than 6 prompts were answered (45). Mplus code for all models can be found: https://osf.io/gnrz7/.

## Results

**Figure 3:**
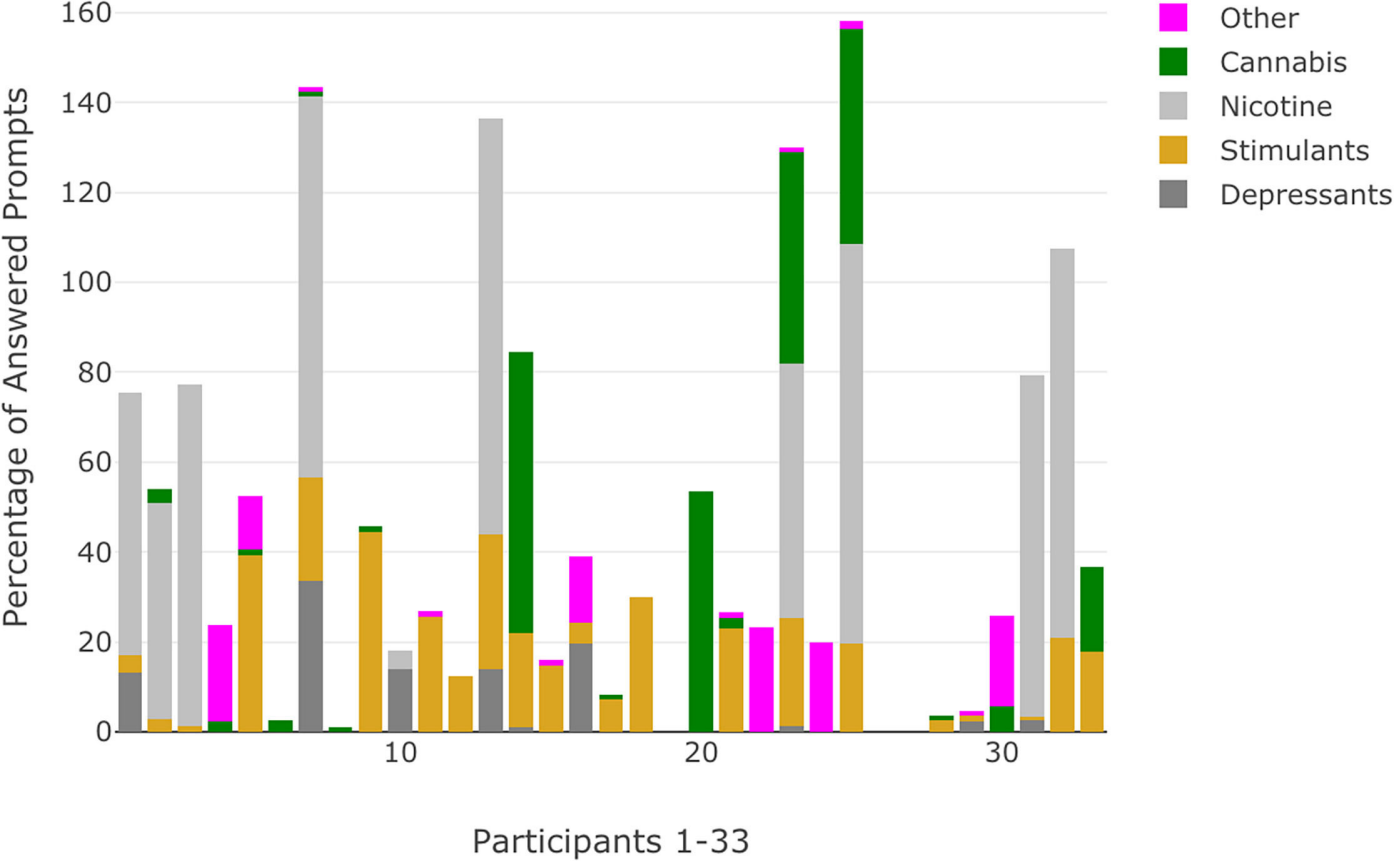
Percentage of answered prompts that indicate substance use categorized by substance use class for each participant. Percentages that exceed 100 reflect participants who indicated use of substances across multiple classes at the same prompt.

Among the 33 participants (see Table 1 for demographic characteristics) included in the analyses, SCID interviews identified 13 (39.4%) that met criteria for a current or lifetime substance use disorder with 4 (12.1%) indicating a lifetime cannabis use disorder, 6 (18.2%) a current cannabis use disorder, 1 (3.0%) a lifetime sedative use disorder, 1 (3.0%) a lifetime stimulant use disorder, and 1 (3.0%) with multiple substance use disorders (cannabis, opioid, stimulant). Data show high rates of compliance to semi-random prompts across CHR (*M* = 99.1 [78.6% of possible prompts], *SD* = 14.5) and EP (*M* = 97.9 [77.7% of possible prompts], *SD* = 19.4) participants. Using ESM data, most participants (*N* = 30, 90.9%) indicated substance use at one prompt or more during the 3-week data collection window; only 3 participants (9.1%) did not indicate any momentary substance use. The majority of participants (*N* = 27, 81.8%) were categorized as having RSU (Table 2). The most common substances included stimulants/caffeine (*N* = 15, 45.5%), products containing nicotine (*N* = 9, 27.3%), depressants (*N* = 4, 12.1%), and cannabis (*N* = 6, 18.2%). No participants indicated use of psychedelics and one participant (3.0%) indicated RSU of sedatives, which was subsequently recoded into the other category, which included four other participants (12.1%) who indicated RSU. Additionally, participants with RSU indicated substance use at ~48% of answered prompts. Substance use patterns reflected pervasive and consistent use for participants with RSU of nicotine (~74% of answered prompts) and moderate for participants with RSU of cannabis (~39% of answered prompts). Visualizations of momentary substance use reflected the percentage of answered prompts by each substance use category, including prompts when multiple substance classes were indicated (Figure 3).

**Table 1:** Demographic characteristics for participants with N (%) reported unless otherwise noted.

	**CHR**	**EP**	**Total sample**
CHR	17 (100)	0 (0)	17 (51.5)
EP	0 (0)	16 (100)	16 (48.5)
Age, mean (SD), range	19.53 (2.9), 16–24	19.63 (2.3), 16–24	19.58 (2.6), 16–24
**Sex assigned at birth**			
Male	7 (41.2)	9 (56.3)	16 (48.5)
Female	10 (58.8)	7 (43.8)	17 (51.5)
**Gender**			
Male	4 (23.5)	8 (50.0)	12 (36.4)
Female	7 (41.2)	6 (37.5)	13 (39.4)
Trans male/Trans man	2 (11.8)	0 (0)	2 (6.1)
Trans female/Trans woman	2 (11.8)	1 (6.3)	3 (9.1)
Genderqueer/Gender non-conforming	2 (11.8)	1 (6.3)	3 (9.1)
**Race and ethnicity**			
White	14 (82.4)	10 (62.5)	24 (72.7)
Hispanic/Latin	2 (11.8)	1 (6.3)	3 (9.1)
Black	1 (5.9)	2 (12.5)	3 (9.1)
Interracial	0 (0)	1 (6.3)	2 (6.1)
Other	2 (11.8)	2 (12.5)	4 (12.1)
**Occupation and education**			
Years of education, mean (SD), range	12.4 (2.6), 10–17	12.2 (1.9), 7–15	12.7 (2.3), 7–17
Student	14 (82.4)	12 (75)	26 (78.8)
Worked full time	4 (23.5)	0 (0)	4 (12.1)
Worked part time	4 (23.5)	6 (37.5)	10 (30.3)
Worked within the last year	4 (23.5)	4 (25)	8 (24.2)
Did not work within the last year	5 (29.4)	6 (37.5)	11 (33.3)
**Parent's education**			
No schooling	0 (0)	1 (3.1)	1 (1.5)
Some high school	2 (5.9)	1 (3.1)	3 (4.6)
Completed high school	9 (26.5)	0 (0)	9 (13.8)
Some college/technical school	6 (17.7)	7 (21.9)	13 (20.0)
Completed college/technical school	6 (17.7)	13 (40.6)	19 (29.2)
Some graduate/professional school	2 (5.9)	0 (0)	2 (3.1)
Completed graduate/professional school	9 (26.5)	9 (28.1)	18 (27.7)
**Living arrangement**			
With family	12 (70.6)	13 (81.3)	25 (75.8)
On own in apartment/dorm	4 (23.5)	1 (6.3)	5 (15.2)
With other(s)	0 (0)	2 (1.3)	2 (6.1)
Other	1 (5.9)	0 (0)	1 (3.0)
**Income**			
< $20,000	3 (17.6)	2 (12.5)	5 (15.2)
$20,000 – $39,999	0 (0)	2 (12.5)	2 (6.1)
$40,000 – $59,999	1 (5.9)	1 (6.3)	2 (6.1)
$60,000 – $99,999	4 (23.5)	1 (6.3)	5 (15.2)
$100,000 or more	2 (11.8)	4 (25)	6 (18.2)
No response/unknown	7 (41.2)	6 (37.5)	13 (39.4)

*CHR, Clinical High Risk; EP, Early Psychosis*.

**Table 2:** Rate of recurrent substance use with experience sampling data characteristics by CHR and EP groups with *N* (%) reported unless otherwise noted.

	**CHR** ***n* = *17***	**EP** ***n* = *16***	***p*-value**	**Total sample** ***n = 33***
Number of valid reports [mean (SD) range]	99.1 (14.5) 72–127	97.9 (19.4) 72–117	0.85	98.5 (16.8) 71–127
RSU any class	14 (82.4)	13 (81.3)	0.93	27 (81.8)
% of prompts that indicate any substance use	44.0	52.1		47.7
RSU Nicotine	5 (29.4)	4 (25.0)	0.78	9 (27.3)
% of prompts that indicate nicotine use	70.8	78.4		74.2
RSU stimulant	8 (47.1)	7 (43.8)	0.85	15 (45.5)
% of prompts that indicate stimulant use	24.6	19.7		23.6
RSU cannabis	2 (11.8)	4 (25.0)	0.32	6 (18.2)
% of prompts that indicate cannabis use	47.4	34.9		39.1
RSU depressant	1 (5.9)	3 (18.8)	0.26	4 (12.1)
% of prompts that indicate depressant use	33.7	15.6		20.1
RSU other	4 (23.5)	1 (6.3)	0.17	5 (15.2)
% of prompts that indicate sedative/other use	26.8	23.6		26.2
RSU One substance class	9 (52.9)	6 (37.8)	0.37	15 (45.5)
% of prompts that indicate any substance use	31.9	40.5		35.3
RSU two or more substance classes	5 (29.4)	6 (37.5)	0.62	11 (33.3)
% of prompts that indicate any substance use	65.9	64.8		65.2
RSU three or more substance classes	4 (23.5)	1 (6.3)	0.17	5 (15.1)
% of prompts that indicate any substance use	72.2	95.3		76.8
Indicated prescription medications	13 (76.5)	15 (93.8)	0.16	28 (84.8)
Indicated medication adherence (≥90% of days)	7 (53.8)	12 (80.0)	0.14	19 (67.9)
NA [mean (SD) range]	3.0 (1.2) 1.1–5.6	2.3 (1.1) 1.1–4.1	0.07	2.7 (1.2) 1.1–5.6
PA [mean (SD) range]	3.5 (1.1) 1.3–5.0	4.2 (1.2) 2.2–6.7	0.09	3.9 (1.2) 1.3–6.7
PSY [mean (SD) range]	2.3 (1.3) 1.0–6.3	1.8 (1.0) 1.0–4.0	0.20	2.1 (1.2) 1.0–6.3

*CHR, Clinical High Risk; EP, Early Psychosis; NA, Negative Affect; PA, Positive Affect; PSY, Psychotic Symptoms; RSU, Recurrent Substance Use*.

*p value reported for Chi square (categorical variables) and t-tests (continuous variables) comparing EP and CHR groups*.

**Figure 4:**
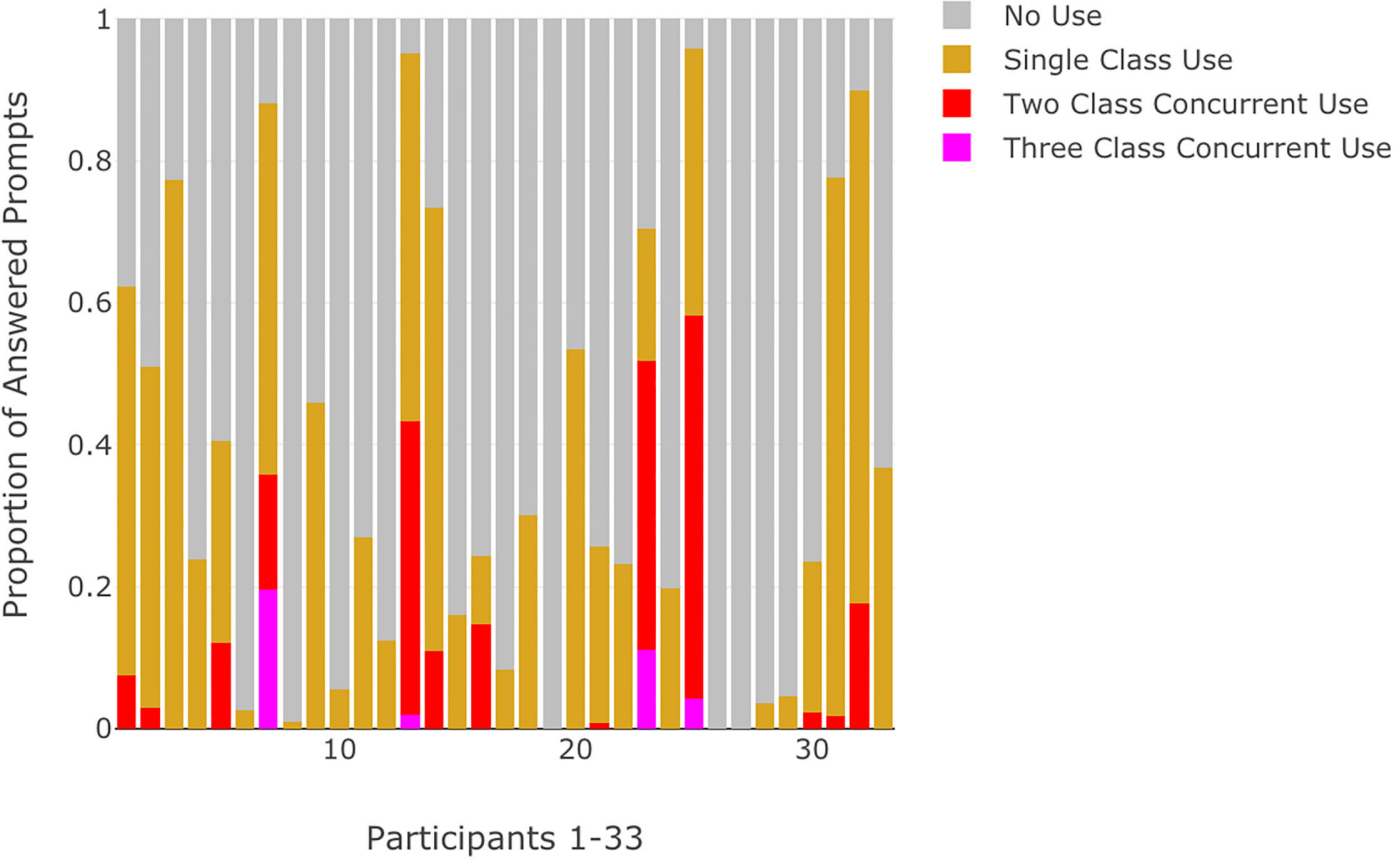
Proportion of answered prompts categorized by no use, single substance class use, or multiple concurrent substance use for each participant.

Approximately half of the participants with RSU indicated use that was limited to a single substance class (*N* = 14, 55.6%). For the remaining participants with RSU, six (22.2%) specified recurrent use for two classes of substances while five participants (18.5%) indicated recurrent use for three or more classes of substances. Visualizations of momentary substance use reflected the proportion of answered prompts with no use, single substance class use, and multiple substance class use (Figure 4). Most participants responded that they were prescribed medication (*N* = 28, 84.8%) with a majority of these individuals indicating that they were adherent at least 90% of the time (*N* = 19, 67.9%). There were no significant differences between the CHR and EP participants for number of answered prompts, rates of RSU, or mean momentary symptom ratings (Table 2). CHR participants showed a trend of higher mean NA ratings (*t* = 1.9, *p* = 0.07) over the 3-week ESM window compared to EP participants. Chi-square and *t*-tests did not achieve appropriate power (0.34–0.54) for observed effect sizes.

### MVAR Model Results

Table 3 provides the posterior median parameter estimates for the MVAR model of substance use (any class), psychotic symptoms, and NA. Results indicated significant autoregressive relationships for NA (β = 0.33, 95% CI: 0.25, 0.41) and psychotic symptoms (β = 0.39, CI: 0.28, 0.49) such that deviations from the person-centered mean at *t-1* predict deviations in the same direction for the subsequent timepoint *t*. Model estimates showed no significant associations between substance use and lagged psychotic symptoms (β = 0.02, CI: −0.01, 0.05), nor with psychotic symptoms (β = 0.01, CI: −0.02, 0.05), or NA (β = −0.02, CI: −0.07, 0.04) at prompt *t*. However, substance use was associated with lagged NA (β = −0.02, CI: −0.05, < -0.00) such that participants who indicated that they had used substances since the previous prompt (e.g., interval between prompt *t-1* and prompt *t*) had experienced lower NA at the previous prompt.

**Table 3:** Posterior median parameter estimates with 95% credible intervals for MVAR model of substance use, psychotic symptoms, and negative affect.

**Parameter**	**Estimate**	**95% Credible interval**
**Intercepts**		
Intercept (NA)	< -0.01	[−0.03, 0.02]
Intercept (PSY)	< -0.00	[−0.02, 0.02]
**Intercept (SU)**	**0.35**	**[0.24, 0.47]**
**Ln Var NA**	**−1.25**	**[−1.57**, **−0.94]**
**Ln Var PSY**	**−2.31**	**[−2.94**, **−1.68]**
**Ln Var SU**	**−2.56**	**[−3.12**, **−2.00]**
**Regression path intercepts**		
**Autoregressive NA**	**0.33**	**[0.25, 0.41]**
**Autoregressive PSY**	**0.39**	**[0.28, 0.49]**
SU on NA	−0.02	[−0.07, 0.04]
**SU on Lag NA**	**−0.02**	**[−0.05**, ** < -0.00]**
SU on PSY	0.01	[−0.02, 0.05]
SU on lag PSY	0.02	[−0.01, 0.05]
NA on lag PSY	−0.09	[−0.23, 0.02]
**PSY on lag NA**	**0.03**	**[0.01, 0.06]**
**NA on PSY**	**0.64**	**[0.46, 0.84]**
**Between–person residual variances**		
Intercept NA	<0.01	[<0.01, <0.01]
Intercept PSY	<0.01	[<0.01, <0.01]
**Intercept SU**	**0.10**	**[0.07, 0.18]**
**Autoregressive NA**	**0.05**	**[0.03, 0.09]**
**Autoregressive PSY**	**0.07**	**[0.04, 0.13]**
SU on NA	<0.01	[<0.01, 0.01]
SU on lag NA	<0.01	[<0.01, <0.01]
SU on PSY	<0.01	[<0.01, 0.01]
SU on lag PSY	<0.01	[<0.01, 0.01]
**NA on lag PSY**	**0.07**	**[0.03, 0.16]**
PSY on NA lag	<0.01	[<0.01, 0.01]
**NA on PSY**	**0.23**	**[0.11, 0.49]**
**Variance NA**	**0.83**	**[0.52, 1.41]**
**Variance PSY**	**3.37**	**[2.18, 5.88]**
**Variance SU**	**2.53**	**[1.60, 4.35]**

*NA, Negative Affect; PSY, Psychotic Symptoms; SU, Substance Use; Ln Var, Natural log of variance estimate; lag, lagged response at prompt t-1*.

*Bold indicates significant parameters*.

When considering the variances (intercepts) of person-specific means over time for each symptom, individuals exhibited more variability around their person-centered mean of NA compared to the variability around their person-centered mean of psychotic symptoms. These estimates were small relative to random effect variances estimates, which suggest the way an individual's momentary responses fluctuate around their person-centered mean are not considerably different across people. In contrast, random effect variances estimates of NA (α = 0.83), psychotic symptoms (α = 3.37), and substance use (α = 2.53) indicated that there are likely distinct differences across people in regards to the way each respective symptom can be explained from the autoregressive and cross-lagged paths estimated in the MVAR model.

The between-level variances for NA and psychotic symptom autoregressive paths had credible intervals of [0.03, 0.10] and [0.05, 0.14], respectively, which suggest that the degree to which psychotic symptoms and NA at prompt *t-1* are associated with psychotic symptoms and NA, respectively, at prompt *t* varies across people. Conversely, the between-level variances for most inter-symptom paths (e.g., substance use associations between lagged or contemporaneous psychotic symptoms/negative affect) had credible intervals between [0.00, 0.01] which suggests little variability across people; the credible interval remained positive as Mplus does not allow negative values. The exceptions included the path between negative affect and lagged psychotic symptoms with a credible interval of [0.03, 0.18] and the contemporaneous path between negative affect and psychotic symptoms [0.11, 0.49]. These variance estimates suggested that there was between-person variability in regards to the degree momentary psychotic symptoms at prompt *t-1* were associated with momentary NA at the subsequent timepoint, prompt *t* and also to the degree negative affect was associated with psychotic symptoms at prompt *t*.

### *N* = 1 Autoregressive (1) Lag Models

Individual autoregressive lag models were estimated for 29 participants, who indicated substance use during at least one answered prompt (an additional participant was excluded due to no variation in psychotic symptoms). Person-level MVAR model diagrams with significant parameters and corresponding credible intervals can be found for each participant within Supplementary Materials (https://osf.io/gnrz7/). Person-level models reflected variable symptom relationships. Of the 29 person-level models estimated, 3 (10.3%) indicated significant substance use and psychotic symptom paths that were not detected in the MVAR model (Participants 2, 7, 30 in Supplementary Materials). All three participants had RSU with nicotine with one of the participants indicating polysubstance use with additional RSU of depressants and stimulant classes. Two (6.9%) person-level models indicated a significant lagged, positive association between substance use and psychotic symptoms. This suggests that higher levels of momentary psychotic symptoms at prompt *t-1* were associated with subsequent substance use that occurred between prompt *t-1* and *t*. Two (6.9%) person-level models indicated a significant positive association between substance use and psychotic symptoms, which indicates that these participants were more likely to exhibit greater levels of psychotic symptoms at prompts when substance use was indicated in the interval between prompt *t-1* and prompt *t*.

## Discussion

The relationship of substance use, including medications and over-the-counter products, to psychotic and mood symptoms during the emergence of schizophrenia and other psychotic-spectrum disorders is complex. Yet understanding this relationship is essential for diagnostic and treatment decision-making, public policy, and systems of service delivery. Much of what is known comes from retrospective self-report or prospective clinician ratings at one or, perhaps, several points over the course of months or years. Less is known about substance use patterns and their potential associations with psychotic and mood symptoms within the daily lives of these adolescents and young adults. The ESM data from individuals with CHR and EP that are reported here provide a new window into these day-to-day patterns with the hope that they can inform the targets and timing of interventions designed to interrupt progression of both substance misuse and serious mental health symptoms.

As expected, a majority of the sample (90.9%) reported use of substances, including illicit, over-the-counter, and medications falling within the selected classes. A majority (*n* = 27, 81.8%) of this sample were characterized as having RSU, defined as substance use occurring during at least 5% of answered prompts. For this group as a whole, 48% of answered prompts reflected consistent momentary use of substances, with those who used nicotine (*n* = 9, 27.3%) and cannabis (*n* = 6, 18.2%) reporting high and moderate frequencies of use (74 and 39% of answered prompts, respectively). Stimulant use (which included caffeine) was the most commonly reported RSU class (*n* = 15, 45.5%), but ESM data reflected less consistent use (23.6% of answered prompts) relative to nicotine and cannabis use. Although rates of use align with previously reported clinical and epidemiological data for CHR and EP samples, these ESM data provide an initial look into the frequency of substance use at the momentary level. No differences were observed between diagnostic groups for RSU rate or momentary mean of NA or psychotic symptoms, but these comparisons were not adequately powered, rendering this finding unreliable.

Given the frequency of momentary substance use observed within this sample, MVAR models were estimated to examine whether substance use was related to lagged or subsequent deviations from person-centered means of psychotic symptoms and NA. We anticipated increased NA to precede substance use and both increased NA and substance use to precede increased psychotic symptoms, consistent with the concept of self-medication. To our surprise, parameter estimates suggested that *lower* levels of NA were associated with subsequent substance use. Although all classes of substances (including prescription medications) are combined into the substance use variable, creating the real possibility that effects cancel each other out, this finding does not support a general theory of self-medication. One possible explanation may be that individuals with CHR or EP engage in substance use when they are in situations (e.g., with peers) that are more positive and less negative. They may also use in an effort to *maintain* lower momentary experiences of NA that occur prior to substance use.

Contrary to a previous ESM study which indicated that cannabis use resulted in increases in hallucinations and decreases in negative affect (31), no significant within-person associations were observed between substance use and subsequent psychotic symptoms. Differences in findings may be due to the current's study design aggregating momentary ratings of nine psychotic symptom items that include both hallucinations and delusions. Inter-symptom relationships may vary based on the specific type of psychotic symptom (hallucinations vs. delusions). Prior work has also noted that potency may moderate cannabis associations to psychosis incidence (46), psychotic episode relapses (47), and positive symptoms (48). Future studies should assess quantity and potency of specific substances and their lifetime use to determine if these factors may moderate experiences of NA or psychotic symptoms in individuals with CHR or EP. Additionally, sample size constraints in the current study limited MVAR variable selection and analysis of specific drug class use. A sensitivity analysis limited to individuals with RSU of nicotine and/or cannabis use (i.e., the two most frequently used substances) indicated a stronger association between nicotine/cannabis use and lagged NA (β = 0.12, CI: −0.20, −0.05). No new significant associations were observed (see Sensitivity Analysis Table at: https://osf.io/gnrz7/).

Furthermore, current study findings differed from previous between-person analyses that have found substance use to be associated with higher levels of psychotic symptoms. While current results may be attenuated by the small sample size, previous work has suggested that within-person associations between cannabis and psychotic symptoms may differ from between-person associations that may be observed longitudinally (21). An important consideration in understanding the mixed findings observed in clinical and epidemiological data, and another advantage of ESM data, is the degree to which there is between-person variability in within-person associations over time (person-level analyses). For one individual, substance use may exacerbate psychotic symptoms while for another, substances may be used as a coping strategy. In the current study, three individual autoregressive lag models indicated positive associations between substance use and psychotic symptoms that were not significant in the MVAR model. While these statistical approaches need to be validated, this type of ideographic analysis is likely to be more useful to personalizing early intervention strategies and to research that disentangles the nature of these relationships.

Of course, the descriptive and exploratory findings must be considered within the context of study limitations. This ESM study was not specifically designed to test associations between substance use and psychotic symptoms and NA; dynamic analyses were exploratory. Individuals with severe substance use expected to interfere with the accurate assessment of other variables were excluded. The small sample and collection of binary data on substances by class restrict our ability to fully understand the day-to-day patterns of specific substance use or disentangle relationships between symptoms and medication, over-the-counter, and illicit substances. In particular, the stimulant class included a combination of legal substances (*e.g*., caffeine products), prescription medications (*e.g*., Adderall), and illicit substances (*e.g*., cocaine). Future work may include multiple questions or non-binary items to assess substance use and include items assessing cravings and the quantity/potency of substances used, or examining use in larger and more homogeneous samples (e.g., only CHR or EP, adolescent or adult).

The MVAR model examined substance use associations across all classes of substance use. These associations are likely multifactorial and expected to differ not only by the specific substance class and specific substance but also by means of ingestion, dose, and potency. Additionally, while analyses accounted for missing data, including non-measurement intervals that occur between response days with fixed interval spacing (2-hours), actual interval spacing between adjacent prompts varied between 1 and 240 min. Modeling of time is an important consideration for ESM studies of substance use, considering that substances differ in terms of pharmacokinetics (i.e., the duration of a specific substance effect) (49). To control for the timing of substance intake, future ESM studies may utilize event-contingent sampling whereby prompts are answered after each instance of substance use (50, 51) in contrast to the semi-random time sampling procedures used in the current study. Finally, analyses are limited to the 3-week ESM data collection window; missing patterns of use and symptom-substance relations that vary episodically or during an acute episode may not have been captured during the study.

Despite these limitations, these data provide new information on the frequency of momentary substance use across important classes of substances, over a meaningful period of time, and in a sample for which properly targeted interventions may have long-lasting effects. Associations between substance use and psychosis differ from common theories that substance use during the course of emerging psychosis is primarily a means of self-medication or that entire classes of substances exacerbate psychosis. However, given the impracticality of truly experimental designs (randomizing individuals to use or not use substances at specific intervals in real life), the analyses demonstrate the potential of statistical modeling of ESM data to increase our understanding of the dynamic substance use and symptom relationships within individuals and across the emergence of and recovery from psychotic disorders. Within-person associations are likely to vary on an individual level, by substance, and over time. Understanding individual patterns over time may be key to disrupting the progression of pathology.

## Data Availability Statement

The raw data supporting the conclusions of this article will be made available by the authors, without undue reservation.

## Ethics Statement

The studies involving human participants were reviewed and approved by Maine Medical Center Research Institute Beth Israel Deaconess Medical Center. Written informed consent to participate in this study was provided by the participants' legal guardian/next of kin.

## Author Contributions

DW and EB drafted the manuscript. KW, RM-G, KE, KJ, KP, and DR critically revised the manuscript. KW, DR, and KP contributed to study conception and design. DW, EB, KW, RM-G, KE, KJ, KP, and DR contributed to acquisition, analysis, and interpretation of data. All authors contributed to the article and approved the submitted version.

## Funding

The National Institute of Mental Health (grant number: R21 MH116240) provided support for KE, KJ, KP, RM-G, and KW with funding for the research activities described in the manuscript.

## Conflict of Interest

The authors declare that the research was conducted in the absence of any commercial or financial relationships that could be construed as a potential conflict of interest.

## Publisher's Note

All claims expressed in this article are solely those of the authors and do not necessarily represent those of their affiliated organizations, or those of the publisher, the editors and the reviewers. Any product that may be evaluated in this article, or claim that may be made by its manufacturer, is not guaranteed or endorsed by the publisher.
